# Culturally competent library services and related factors among health sciences librarians: an exploratory study

**DOI:** 10.5195/jmla.2017.203

**Published:** 2017-04

**Authors:** Misa Mi, Yingting Zhang

## Abstract

**Objective:**

This study investigated the current state of health sciences libraries’ provision of culturally competent services to support health professions education and patient care and examined factors associated with cultural competency in relation to library services and professional development.

**Methods:**

This was a cross-sectional study. Data were collected with a survey questionnaire that was distributed via SurveyMonkey to several health sciences librarian email discussion lists.

**Results:**

Out of 176 respondents, 163 reported serving clients from diverse cultural backgrounds. Various services were provided to develop or support initiatives in cultural competency in health professions education and patient care. A considerable number of respondents were unsure or reported no library services to support initiatives in cultural competency, although a majority of respondents perceived the importance of providing culturally competent library services (156, 89.1%) and cultural competency for health sciences librarians (162, 93.1%). Those who self-identified as nonwhites perceived culturally competent services to be more important than whites (*p*=0.04). Those who spoke another language in addition to English had higher self-rated cultural competency (*p*=0.01) than those who only spoke English.

**Conclusions:**

These findings contribute to our knowledge of the types of library services provided to support cultural competency initiatives and of health sciences librarians’ perceived importance in providing culturally competent library services and cultural competency for health sciences librarians. The results suggest implications for health sciences libraries in fostering professional development in cultural competency and in providing culturally competent services to increase library use by people from a wide range of cultures and backgrounds.

## INTRODUCTION

The Association of College & Research Libraries (ACRL) developed a set of diversity standards for academic libraries [1]. These standards have served as a framework for providing services to diverse populations and maintaining a diverse workforce. Health sciences librarians interact with and serve students, faculty, health care providers, and patients from culturally and linguistically diverse backgrounds. To provide resources and services that enhance the provision of culturally effective health care, it is essential for health sciences librarians to develop cultural competency [2]. Cultural competency is viewed as one’s ability to understand the needs of people whose culture and language are different from one’s own and to interact effectively with people from different cultures [3, 4]. Cultural competency is also referred to as:

The ability to recognize the significance of culture in one’s own life and in the lives of others; and to come to know and respect diverse cultural and background characteristics through interaction with individuals from diverse linguistic, cultural, and socioeconomic groups; and to fully integrate the culture of diverse groups into services, work, and institutions in order to enhance the lives of both those being served by the library profession and those engaged in service. [5]

According to the 2010 US Census, the population increase between 2000 and 2010 was mainly in nonwhite and Hispanic or Latino ethnic groups [6]. More than half of all Americans are projected to belong to a minority group (any group other than non-Hispanic whites alone) by 2044, and nearly one in five of the nation’s total population is projected to be foreign born by 2060 [7]. As the country becomes more diverse, health care providers will increasingly see patients with a broad range of perspectives regarding health, often shaped by their social or cultural backgrounds [8]. These backgrounds contribute to social determinants that influence one’s ability to achieve good health and are closely linked with different health outcomes or health care disparities [9]. Furthermore, these disparities are exacerbated by the conscious and unconscious biases of health care professionals as well as patients’ fear of bias, which gives rise to a mistrust of doctors [10]. Cultural competency is linked to reducing or eliminating racial or ethnic disparities in health care. Elimination of health care disparities is one of the overarching goals of Healthy People 2020 [11]. Initiatives such as cultural competency education have been proposed as a strategy to prepare the future health care workforce to care for diverse patient populations [8, 12–14].

The Joint Commission has developed a roadmap for hospitals to incorporate cultural competency into their organizations [15]. The standards of the Liaison Committee on Medical Education (LCME) mandate that a medical educational program provide learning experiences to develop students’ cultural competency and prepare them for delivering culturally competent care for increasingly diverse patient populations [16]. In the same vein, the American Nurses Association has stated its position on the importance of cultural competency at all levels of nursing practice [17], and the American Association of Colleges of Nursing has included cultural competency as part of outcome competencies for graduates [18].

In addition to emerging regulatory and accreditation pressures for undergraduate and graduate medical education and nursing education, changing demographics of the nation, funding opportunities, and increasing diversity of patients, students, and faculty have served as key drivers of cultural competency [8]. Health sciences libraries, which constitute part of the educational learning environment, are indispensable to health professions education (education of health care professionals) and quality patient care alike. Cultural competency needs to be an integral part of service delivery, workforce equity, and leadership development [19]. It is important for health sciences libraries to develop resources, services, and educational programs in response to the call for health care professionals and educators to take important and unique roles in academic medicine to help reduce health inequity and disparities [20] and to meet information needs in cultural competency education for health professions students.

This study was conducted to: (1) investigate how health sciences libraries provide services to support initiatives in cultural competency in health professions education and patient care, (2) examine health sciences librarians’ attitudes toward cultural competency in relation to health libraries services and professional development, and (3) identify any demographic characteristics associated with these attitudes.

## METHODS

This exploratory study with a cross-sectional design was conducted with a survey questionnaire. It was developed and piloted on a small sample of health sciences librarians to enhance its content validity before it was distributed to email discussion lists to recruit participation. Feedback was gathered and incorporated into revisions of the questionnaire (supplemental appendix). With fifteen items, the questionnaire obtained information on demographics of study participants, types of clients served, services provided to support cultural competency initiatives in health professions education and patient care, barriers to providing culturally competent services, and librarians’ attitudes toward cultural competency. This study was conducted with approval from both Oakland University’s Institutional Review Board (IRB) and Rutgers University’s Health Sciences IRB.

The online survey was administered using SurveyMonkey. The link to the survey questionnaire was distributed to a convenience sample of health sciences librarians who subscribed to several email discussion lists, including MEDLIB-L, the Health Sciences Library Association of New Jersey (HSLANJ) email list, and the email list of the Michigan Health Sciences Libraries Association. The survey was run for 6 weeks from November 6, 2015, to December 18, 2015. Survey data were imported to SPSS for data analysis. Data were analyzed using both descriptive statistics (means, standard deviations [SD]) and inferential statistics (Tukey’s tests) for pairwise comparisons. The significance threshold was set at <0.05.

One purpose of the survey was to investigate whether respondents’ demographic information (i.e., type of library, age, gender, race, and language spoken; questions [Q] 1–5) was related to their perceived importance of providing culturally competent library services (Q10), perceived importance of cultural competency for health sciences librarians (Q11), or self-rated level of cultural competency (Q13). Responses to Q10, Q11, and Q13 were treated as continuous numbers and reported on a scale of 1 to 5 (5=extremely important and 1=not important). The mean and SD of these responses were broken down for each of the response possibilities for Q1–5. Not all respondents answered all questions.

## RESULTS

### Demographic information

Out of 176 respondents, 73 were from health sciences or medical libraries in academic settings, 78 were from hospital settings, and 25 were from general academic, special, consumer health, and public libraries. A large number of respondents (n=117, 67.3%) were aged 50 years or older. There were more females (n=149, 85%) than males (n=26, 15%), representing white (n=138, 79.8%), Asian (n=17, 9.8%), black or African American (n=14, 8.1%), Hispanic or Latino (n=4, 2.3%), American Indian or Alaska Native (n=1, 0.6%), and Native Hawaiian or other Pacific Islander (n=1, 0.6%) races. Out of 135 respondents, 48 (36%) spoke more than 1 language.

### Library services to support cultural competency

Regarding the types of library clientele served, the primary clientele comprised students (n=153, 88.4%) and staff (n=153, 88.4%), followed by faculty (n=134, 77.5%), administration (n=125, 72.3%), community (n=89, 51.4%), and patients (n=74, 42.8%). Other clientele included residents, physicians, nurses, and hospital staff. The majority of respondents (n=163, 93%) reported that they served clients from diverse cultural backgrounds, whereas 7 were unsure and 5 did not. Leading the types of library services provided to support initiatives in cultural competency were resource collections (n=97, 58.1%), followed by resource guides (n=46, 27.5%), web pages (n=35, 21%), outreach programs (n=32, 19.2%), cultural competency training (n=17, 10.2%), and course guides (n=17, 10.2%). However, 44 (26.3%) respondents reported no services were provided for that purpose, and 14 (8.4%) were unsure. A few other services included cultural competency in-service training for library staff, library instruction on finding information on transcultural nursing, a global initiatives project connecting librarians with scholars from other countries, interpreter assistance, book discussion, and library liaison with patient care staff and research personnel from a diverse cultural background.

Regarding barriers to providing culturally competent library services, 80 (46.5%) respondents reported no barriers; 50 (29.1%) faced barriers; and 42 (24.4%) were unsure. Respondents also shared their views on certain barriers when providing culturally competent services, including but not limited to lack of linguistically or culturally competent staff and librarians, inadequate budget, time constraint, and lack of space. Some respondents offered comments such as the following:

“I have many materials in my collection that address cultural competency for health professionals and support materials for a course in cultural competency. However, I make no effort to provide materials in languages other than English since all our students are required to have an undergraduate degree from a US institution.”

“It’s the whole we don’t know what we don’t know problem. If people perceive a barrier, it may not be something about which we are ever made aware.”

### Health sciences librarians’ cultural competency perceptions

Most respondents (n=156, 89.1%) perceived the importance of providing culturally competent library services, whereas 16 (9.1%) were unsure and 3 (1.7%) perceived it as unimportant. A majority of respondents (n=162, 93.1%) perceived cultural competency for health sciences librarians as important, whereas 9 (5.2%) were unsure and 3 (1.7%) perceived it as unimportant. Regarding any previous course work or training in cultural competency, 81 (46.3%) respondents reported to have training, whereas 87 (49.7%) indicated no past training experience and 7 (4.0%) were unsure. In self-rating their own level of cultural competency on a 5-point scale from highly competent (5) to not competent (1), 76 (43.9%) chose a rating of 4, 60 (34.7%) chose 3, 18 (10.4%) chose 2, 16 (9.3%) chose 5, and 3 (1.7%) chose 1. When asked about their interest in taking a continuing education (CE) course in cultural competency, 139 respondents (79.4%) would consider taking one, whereas 23 (13.1%) were unsure and 13 (7.4%) would not consider taking one.

### Influence of demographic characteristics on health sciences librarians’ perceptions of cultural competency

There were no significant differences in how librarians from different types of libraries perceived the importance of providing culturally competent services (*p*=0.51), the importance of cultural competency for health sciences librarians (*p*=0.72), or their own cultural competency (*p*=0.10). Librarian age also had no significant influences on these perceptions (*p*=0.21, *p*=0.19, and *p*=0.31, respectively). Although there were no significant gender differences in perceptions of the importance of cultural competency for health sciences librarians (*p*=0.18) or their own cultural competency (*p*=0.14), female librarians placed higher importance on providing culturally competent library services than male librarians (mean: 3.93 versus 3.5, *p*=0.048). Also, nonwhite librarians placed higher importance on providing culturally competent library services than white librarians (mean: 4.20 versus 3.80, *p*=0.04), although there were no significant race differences in other cultural competency perceptions. Those who spoke another language in addition to English rated their own level of cultural competency higher than those who only spoke English (mean: 3.79 versus 3.41, *p*=0.01). Respondents from hospital settings would more likely consider taking a CE course in cultural competency than those from other library settings (*p*=0.02). Compared with respondents who would not consider taking a CE course in cultural competency, those who had such an intention placed higher importance on providing culturally competent library services (mean: 3.96 versus 3.08, *p*=0.003) and on cultural competency for health sciences librarians (mean: 4.05 versus 3.38, *p*=0.02).

## DISCUSSION

The results of this explorative study offered useful insights into cultural competency in relation to health sciences librarianship. These findings help fill the gap in the literature on the subject, point to needs in cultural competency training, and shed light on certain personal characteristics associated with what librarians can do to contribute to creating culturally competent institutions.

A large number of survey respondents identified themselves as white, suggesting that the current racial and ethnic makeup of the health sciences librarian workforce has not changed substantially since the American Library Association reported “white” as the racial/ethnic category selected by an overwhelming majority of respondents in membership surveys conducted in the past [21, 22]. The authors also noted that a considerable number of respondents were in their fifties or older, indicating an anomalous distribution of respondents in the Baby Boomer generation. Therefore, it appears that the demographics of the health sciences librarian workforce are still lacking in diversity based on race and age, despite a demographic trend of increasing ethnically and racially diverse populations in the United States [6, 7, 23] and an aging library profession that has been well documented in earlier reports [22, 24–26]. This lack of diversity should propel librarians to “look critically at our culture, our practices, and our assumptions, and investigate what it is about ourselves and our profession that is preventing underrepresented people from being able to, or even wanting to, enter and stay” [27].

A considerable number of respondents were unsure or reported no library services were provided to support initiatives in cultural competency. In providing culturally competent library services, libraries faced various barriers such as lack of linguistically or culturally competent staff and librarians, inadequate budget, and time constraints. Other barriers included a lack of awareness or “the whole we don’t know what we don’t know problem,” as one survey respondent commented. Respondents’ certain personal factors might affect their attitudes toward cultural competency and perceived importance and value of providing culturally competent library services. The authors found that females were more likely to perceive the importance of providing culturally competent library services than males. However, as there were only 26 male respondents versus 149 female respondents, the gender difference in perceived importance and value of cultural competency for librarians and culturally competent library services warrants further research.

Another finding of this study is that nonwhite librarians were more likely to perceive the importance of providing culturally competent library services than white librarians, suggesting that race may be a determinant of one’s attitude toward the importance of providing culturally competent library services. A possible interpretation for the difference in perceptions is that nonwhite librarians may be more sensitive to and aware of barriers and disparities encountered in their lives or at work. This finding was in accordance with previous research indicating that race is a significant contributing factor to differences in perceived cultural competency [28, 29]. Therefore, it is important for librarians to recognize the need for developing cultural competency that would likely enhance their practice of librarianship and help them better address the social, linguistic, and academic needs of culturally diverse individuals. By providing culturally competent services and programs, health sciences librarians can better meet these needs and increase library use by people from a wide range of cultures and backgrounds. When developing health sciences librarians’ cultural competency, it would be useful to take into consideration special training needs and strengths in specific groups and develop targeted educational interventions to fully engage different groups in learning about and appreciating different cultures.

We also found that those who spoke another language in addition to English rated their own levels of cultural competency higher than those who only spoke English. Those with the ability to speak another language might have an advantage of better understanding a given culture, which could lead to higher levels of cultural awareness and sensitivity. Humans learn a culture through language. Language is a reflection or mirror of its culture or cultural idiosyncrasies [30] and is considered one of the most important differences between cultures [31] and a primary means by which a culture expresses its beliefs, values, norms, and worldviews [32]. Language is also one of the greatest barriers in a cross-cultural situation. Every culture is permeated with unique forms of words, types of conversation, and forms of polite usage, which are thought to be appropriate and manifest different cultural patterns [2]. The study’s findings have implications for health sciences librarians who work with users from multicultural and multilingual backgrounds. Culturally competent librarians should regard the ability to speak a second language as an asset that demonstrates greater cognitive ability [33], rather than a deficiency [5]. It would be worthwhile for librarians to develop awareness and knowledge of language differences (which does not require an ability to speak that language) that are reflected in verbal and nonverbal communication processes and norms for effective cross-cultural interactions with and service provision for users from different backgrounds.

The majority of respondents expressed a willingness to take a CE course in cultural competency, indicating an interest in professional development in cultural competency. It was not surprising that those who were interested in taking a CE course tended to consider it more important to provide culturally competent library services and to develop cultural competency for health sciences librarians than those who were not interested in taking a CE course.

Another interesting finding was that respondents from hospital settings were more likely to consider engaging in professional development in cultural competency than those at other library settings. Today, the health care environment is going through rapid changes, and health care organizations are challenged to deliver culturally competent care for increasingly diverse patient populations. Hospital libraries not only meet the information needs of health care providers from diverse cultural backgrounds, but also are called on to serve diverse patients through their consumer health collections. The patient care–centered work environment might account for the difference in hospital librarians’ views on providing culturally competent library services and developing cultural competency for health sciences librarians. Thus, it is necessary to consider the different professional development needs of health sciences librarians from different settings when developing and offering CE opportunities.

### Implications for practice and research

The mandate for cultural competency in health professions education and the growing attention to and the influence of cultural competency on patient outcomes and health care disparities afford abundant opportunities for health sciences librarians to play an important role in taking the initiative in developing, promoting, and supporting programs in cultural competency in health professions education and patient care. It is advisable for health sciences libraries and health information professional organizations to embody diversity in the health sciences library workforce by identifying and implementing strategies to recruit and retain new graduates or librarians from underrepresented groups for health sciences libraries. Educational offerings for professional development in cultural competency need to equip health sciences librarians with the knowledge, skills, and attitude necessary for working in diverse health care teams and providing resources, services, and programs that contribute to creating culturally competent libraries, culturally competent institutions, and most importantly, culturally competent health care. A line of research that investigates cultural competency in the practice of health sciences librarianship would help determine what impact library initiatives have and how professional development in cultural competency enhances health sciences librarians’ ability to meet the information needs of a growing population of diverse library users and to competently address issues of disparities among diverse populations.

Health information professionals are faced with the challenge of working and interacting with a growing population of individuals from diverse cultural, linguistic, social, and economic backgrounds. The findings gleaned from this study provide a baseline for future studies of how cultural considerations in library services and programs affect library use by diverse populations. The results also serve as a needs assessment for developing future cultural competency training to enhance librarians’ abilities to deal with diverse cultures and to improve their knowledge and understanding of cultural differences and related issues. As knowledge workers, health sciences librarians have unique skills and expertise. The development of their cultural competency will augment their capacity to help empower “the diverse human talents of the most diverse nation on earth” [34].

### Limitations

This study is subject to several limitations inherent in the study design. First, survey data were collected from individuals who subscribed to certain email discussion lists for health sciences librarians and chose to participate in the survey and might not adequately represent the entire health sciences librarian population. Second, study participation was voluntary and participants were all self-selected, which can lead to biased responses. Third, our results might have been influenced by social desirability bias, as respondents might have answered certain questions in a manner that they thought would be viewed favorably by others. Finally, the survey relied on respondents’ self-reports, which is subject to personal or recall bias. Their impressions and memories might not accurately reflect actual types of library services provided, barriers encountered, or training experience in cultural competency.

## Supplemental File

Appendix Culturally competent library services survey
