# Supplementary material for: Culturally competent library services and related factors among health sciences librarians: an exploratory study
Source: J Med Libr Assoc. 2017 Apr;105(2):132–8. doi: 10.5195/jmla.2017.203 (PMC5370603; doi:10.5195/jmla.2017.203)
Supplement: Appendix [file jmla_apr17_mi_app.pdf]

JMLA Journal of the Medical Library Association

SC

Supplemental content to
J Med Libr Assoc. Apr;105(2): dx.doi.org/10.5195/jmla.2017.203
www.mlanet.org
© Mi, Zhang 2017

Culturally competent library services and related factors among health sciences librarians: an exploratory study

Misa Mi, PhD, MLIS, AHIP; Yingting Zhang, MLS, AHIP

APPENDIX

Culturally competent library services survey

Email recruitment message

As the US population is becoming increasingly diverse, are health sciences libraries and librarians aware of and/or providing services to meet the different needs of our diverse users? We are conducting a survey to examine what types of library services are provided to support initiatives in cultural competence in health professions education and patient care and how librarians perceive cultural competence.

We are writing to invite you to participate in the survey that may take approximately five minutes to complete. The survey is open and will close by [date]. Participation is voluntary; no private or personal information will be collected. Clicking on the link below constitutes your consent for participation.

Survey link: https://www.surveymonkey.com/r/cultural_competence_librarians

Thank you for making time to respond to the survey! If you have any questions regarding the survey or would like to get a copy of the survey results, please contact Yingting Zhang at yzhang@rutgers.edu or Misa Mi at mi@oakland.edu.

Survey

1. Type of your current library:
   a. Hospital library
   b. Academic health sciences library
   c. Corporate/special library
   d. Consumer health library
   e. Public library
   f. Other. Please specify ______________________________

2. Your age:
   a. 18–29
   b. 30–39
   c. 40–49
   d. 50–59
   e. >60

SC
Supplemental content to
J Med Libr Assoc. Apr;105(2): dx.doi.org/10.5195/jmla.2017.203
www.mlanet.org
© Mi, Zhang 2017

3. Your gender:
   a. Male
   b. Female
   c. Other

4. Select one or more of the following races:
   a. American Indian or Alaska Native
   b. Asian
   c. Black or African American
   d. Hispanic or Latino
   e. Native Hawaiian or Other Pacific Islander
   f. White

5. Language you speak:
   a. English only
   b. Other(s). Please specify ____

6. What is your library's primary clientele? Select all that apply.
   a. Faculty
   b. Students
   c. Patients
   d. Staff
   e. Administration
   f. Community
   g. Other(s). Please specify ____

7. Do you serve clients with diverse cultural backgrounds?
   a. Yes
   b. No
   c. Unsure

8. What kind of services do you provide to develop or support initiatives in cultural competency in health professions education or patient care?
   a. Resource collections
   b. Outreach programs
   c. Resource guides (e.g., LibGuides)
   d. Course guides
   e. Cultural competence training
   f. Web pages
   g. Other(s). Please specify ____
   h. None

9. Do you face any barriers to providing culturally competent library services? If yes, please specify.

JMLA Journal of the Medical Library Association

SC
Supplemental content to
J Med Libr Assoc. Apr;105(2): dx.doi.org/10.5195/jmla.2017.203
www.mlanet.org
© Mi, Zhang 2017

10. How important is it to provide culturally competent library services?
    a. Extremely important
    b. Very important
    c. Important
    d. Not sure
    e. Not important

11. How important is cultural competence for health sciences librarians?
    a. Extremely important
    b. Very important
    c. Important
    d. Not sure
    e. Not important

12. Have you received any course work/training in cultural competence?
    a. Yes
    b. No

13. Please rate your own level of cultural competence by picking a number from the scale.
    Not competent 1 2 3 4 5 Highly competent

14. Would you consider taking a CE course on cultural competence if it is offered to health sciences librarians?
    a. Yes
    b. No
    c. Unsure

15. What are your suggestions for improving health sciences librarians' cultural competence?
